# Direct contact of fermented rice bran beds promotes food-to-hand transmission of lactic acid bacteria

**DOI:** 10.1093/femsle/fnad120

**Published:** 2023-11-13

**Authors:** Ryo Niwa, Dominique Chen, Young ah Seong, Kazuhiro Jo, Kohei Ito

**Affiliations:** BIOTA Inc., Tokyo, 101-0022, Japan; Graduate School of Medicine, Kyoto University, Kyoto, 606-8501, Japan; Faculty of Letters, Arts and Sciences, Waseda University, Tokyo, 162-8644, Japan; Faculty of Design & Engineering, Hosei University, Tokyo, 162-0843, Japan; Faculty of Design, Kyushu University, Fukuoka, 815-8540, Japan; BIOTA Inc., Tokyo, 101-0022, Japan

**Keywords:** fermentation, microbiome, *Nukadoko*, lactic acid bacteria, food preservation

## Abstract

The skin microbiome, which varies widely between individuals, plays a crucial role in human health. It also interacts with the environment in various ways, including during the preparation of fermented food. *Nukadoko* is a pickle and traditional fermented food in Japan that utilizes lactic acid bacteria to ferment vegetables. When preparing or maintaining *Nukadoko*, it is mixed with bare hands. Despite the known interaction between *Nukadoko* and human skin, no studies have explored its impact on *Nukadoko* quality or skin microbiome changes. This study examines these effects during *Nukadoko* maintenance. Three participants were asked to stir commercially available late-stage *Nukadoko* for 14 days and not stir it for the remaining 14 days to examine microbial settlement and shedding. Microbiome analysis was performed on human skin and *Nukadoko*. We found that microorganisms from rice bran beds can temporarily settle on human skin but are shed quickly. Stirring rice bran beds by hand may have short-term effects on the skin microbiome. This study provides insights into the communication between human and food microbiomes in traditional Japanese fermented foods.

## Teaser

The study investigates the interaction between human skin microbiome and Nukadoko, a Japanese fermented food. Findings suggest short-term effects on skin when stirring rice bran beds by hand.

## Introduction

Fermentation is a phenomenon used for food preservation carried out by microorganisms. Among fermented foods, pickles, flavored by fermenting vegetables, are produced in food industries worldwide. ​Fermentation improves food preservation and aids in the development of its aroma, flavor, and texture. ​Lactic acid bacteria play a primary role in fermentation, specifically in the homofermentation and heterofermentation of lactic acid. Bacteria that are generally undesirable for food preservation, such as gram-positive bacteria, are vulnerable to low pH. These bacteria carry out fermentative production under anaerobic conditions to induce the growth of lactic acid bacteria and production of lactic acid (Voidarou et al. 2020). This type of fermentation is commonly observed in dairy products and in fermented vegetables (Ashaolu and Reale 2020).

*Nukadoko*, a traditional Japanese fermented food (Nakayama et al. 2007), is a rice bran bed that ripens pickles (*Nukazuke*). The traditional and predominantly manual method of preparing *Nukadoko* is to add salt water to the rice bran, knead it well, and then add vegetables to the rice bran bed for natural fermentation in the presence of lactic acid bacteria (Sakamoto et al. 2011; Ono et al. 2014). *Nukadoko* produced in this manner has a variety of microorganisms containing Gram-positive bacteria, Gram-negative bacteria, and yeast (Ono et al. 2014). Recently, the addition of fermentation starters, such as long-aged or commercially available *Nukadoko*, which allows easier and more stable preparation and maintenance of the bed, has become the mainstream method (Sakamoto et al. 2011).

The microbial composition of *Nukadoko* and surfaces of pickled vegetables has been investigated through massively parallel sequencing to identify 16S ribosomal RNA (16S rRNA) amplicon sequences (Nakayama et al. 2007; Sakamoto et al. 2011; Ono et al. 2014, 2015; Sawada et al. 2021). Pyrosequencing-based analysis revealed the microbial dynamics of *Nukadoko* created in the laboratory with 16 different long-term aged bran beds as fermentation starters (Sakamoto et al. 2011). *Nukadoko* of different origins, in combination with fermentation starters, showed a variety of microbial compositions. Another study showed that the microbial diversity of *Nukadoko* with added spices, such as Japanese peppers and red peppers, differed because of the effect of secondary metabolites in spices (Ono et al. 2015). *Nukadoko* from different manufacturers has also been reported to contain different microbiomes (Ono et al. 2014; Sawada et al. 2021). Furthermore, diversity in organic and amino acids, which is influenced by microbiome variations in pickled vegetables (Sawada et al. 2021), significantly affects flavors.

However, for maintaining optimal microbial communities in *Nukadoko*, the rice bran bed requires stirring with bare hands either daily or every few days. The human skin is inhabited by various microorganisms that can affect fermentation (Byrd, Belkaid and Segre 2018). Previous studies have identified human skin-associated *Staphylococcus* in *Nukadoko* at an early stage of preparation (Ishizaki et al. 2001). However, no studies have examined how the skin microbiome, which varies widely from individual to individual, affects the quality of *Nukadoko*. Conversely, *Nukadoko* can contain microorganisms that may benefit the human skin. The effects of continued exposure to *Nukadoko* on the microbial composition of the human skin have never been thoroughly evaluated.

We used 16S rRNA amplicon sequencing to evaluate the effects of interaction between *Nukadoko* and the human skin microbiome during *Nukadoko* maintenance. Three anonymous participants maintained a commercially available *Nukadoko* at a late stage for 30 days. Shared amplicon sequencing variants (ASVs) were computed to identify microorganisms transmitted from *Nukadoko* to human skin. This study sheds light on the human–food microbiome interaction in traditional Japanese fermented foods.

## Materials and methods

### Ethics

The study protocol was approved by the local ethics research committee of Waseda University (Ethics Review Procedures Concerning Research with Human Participants; application number: 2021–423; approved on February 7, 2022). All procedures were conducted according to the ethics committee's guidelines and regulations. All participants provided written informed consent before participating in the study.

### *Nukadoko* maintenance and sample collection

**Figure 1.**
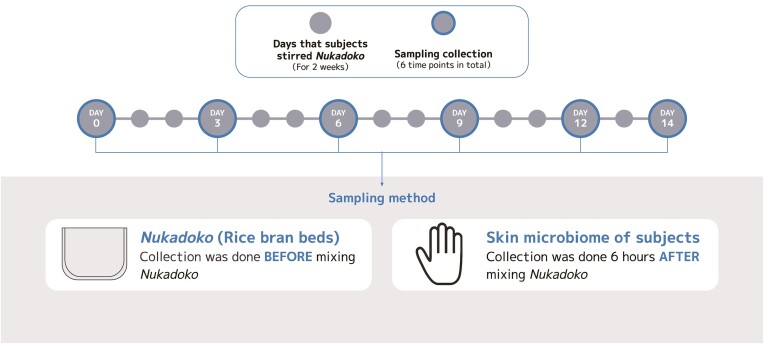
Study workflow. Monitoring was performed for two weeks, and *Nukadoko* and skin samples were collected as a pair. *Nukadoko* samples were collected with plastic spoons in the first 2 weeks. Palm swabbing for skin microbes was done in parallel. A sampling of skin microbiome on days 15 to 29 was done without stirring *Nukadoko* to measure the speed of bacterial shedding.

The study participants were healthy volunteers recruited from acquaintances (N = 3); all were Japanese nationals, of which two were female, and one was male. The study was conducted in Tokyo, Japan, in February and March, 2022. The participants were given commercially available *Nukadoko* at the late stage and were asked to stir it for 14 days and not stir it for the remaining 14 days to examine microbial settlement and shedding on the skin. Participants were asked to turn over *Nukadoko* from the bottom, mixing it thoroughly. This mixing was repeated 2 or 3 times, with the whole session lasting about 3 min. We did not restrict participants from using of soap or ethanol in this study after mixing sessions*. Nukadoko* samples were collected on days 0, 3, 6, 9, 12, and 14 using individually wrapped disposable plastic spoons. Skin microbiome samples were collected on days 0, 3, 6, 9, 12, 14, 15, 18, 21, 24, 27, and 29 by swabbing the palm for 3 min using a sterile cotton-tipped swab (ESwab™; Copan Diagnostics, Brescia, Italy). Swabs were stored in tubes with Liquid Amies Medium solution. (Copan Diagnostics, Brescia, Italy). Both sample types were immediately frozen and stored at −20°C until DNA extraction. The study workflow is illustrated in Fig. 1. The sampling duration for each *Nukadoko* and skin microbiome sample was at least 6 h.

### Total DNA extraction and high-throughput sequencing

Samples were treated with 750 µL of lysis buffer from the GenFind V2 DNA extraction kit (Beckman Coulter, Indianapolis, IN, USA). The suspension was vortexed for 10 min, heat-treated at 100°C for 10 min, and centrifuged for 5 min at 20 000 g. The supernatant was then mixed with EZ beads (AMR, Tokyo, Japan), and DNA was fragmented using the MM-400 unit (Retsch, Haan, Germany) at a maximum speed for 3 min. The remaining DNA purification steps were performed using the abovementioned GenFind V2 DNA extraction kit (Beckman Coulter), according to the manufacturer's protocol. DNA was eluted with 80 µL of nuclease-free water; using the KAPA HiFi HotStart ReadyMix (Roche, Basel, Switzerland) (Caporaso et al. 2011; Klindworth et al. 2013) and specific primers (341F: 5′-TCGTCGGCAGCGTCAGATGTGTATAAGAGAGACACCTACGGGNGGCWGCA G-3′) and 806R (5′-GTCTCGTGGGCTCGGGAGATGTGTATAAGAGACAGGACTACHVGGGTATCT AATCC-3′), the V3–V4 region of the 16S rRNA gene was amplified. The thermal conditions were 95°C for 3 min, followed by 32 cycles at 95°C for 30 s, 55°C for 30 s, and 72°C for 30 s, with a final extension at 72°C for 5 min. DNA samples, library preparation, and amplicon sequencing were performed using 300-bp paired-end sequencing on the Illumina MiSeq platform (Illumina Inc., San Diego, CA, USA) at GenomeRead Inc. (Kagawa, Japan).

### Microbiome analysis

Microbiome analysis was performed as previously reported (Ito et al. 2023). Briefly, raw FASTQ files were imported into the QIIME2 platform (2022.8) as qza files (Bolyen et al. 2019). Denoising and read quality control were performed using the QIIME dada2 denoise-paired function, and reads were classified into ASVs (Callahan et al. 2016). We used 269 nt for-p-trunc-len-f and 255 nt for-p-trunc-len-r. The SILVA database's SSU 138 (https://www.arb-silva.de/documentation/release-138/) was used with the QIIME feature-classifier classification scikit-learn package for taxonomic assignment (Quast et al. 2012; Bokulich et al. 2018). ASVs classified as chloroplast, mitochondria, or unassigned were excluded from subsequent statistical analysis. Subsampling is a common method for inferring microbiome differences between samples and is a suitable analytical approach for analyzing new datasets. To evaluate the effect of sequence read counts on microbiome diversity assessment, we plotted changes in the Shannon index over a range of read counts from 0 to 10 000, using rarefaction curves.

### Custom database for taxonomic assignment

The classifier database used in this study was made from Silva release 138.1 SSU 99% (www.arb-silva) (Quast et al. 2012). Database curation was performed using REference Sequence annotation and CuRatIon Pipeline (RESCRIPt) following the developers’ recommended parameters (Robeson et al. 2021). Briefly, RESCRIPt removed low-quality sequences (sequences containing > 5 or more ambiguous bases or homopolymers of ≥ 8 bases) and filtered lengths (archaea [16S rRNA] ≥900 bp, bacteria [16S rRNA] ≥1200 bp, and eukaryotes [18S rRNA] ≥1400 bp). Additionally, deduplication of reads was performed in the Uniq mode. We then created the scikit-learn naive Bayes classifier using the QIIME2 plugin (feature classifier) (Bokulich et al. 2018).

### Calculation of shared ASVs

We defined shared ASVs as ASVs shared by > 1% of both datasets (*Nukadoko* and skin) in this study. When *Nukadoko* was touched for the first two weeks, data from *Nukadoko* and skin from the same day were used as pairs; when the bran was not touched for the next two weeks, data from *Nukadoko* from the last day and each skin microbiome data were used as pairs. The calculation was conducted using R (version 4.2.1) and phyloseq (version 1.40.0) (McMurdie and Holmes 2013) or the custom python code (q2-shared_asv v0.2.0, https://github.com/biota-inc/q2-shared_asv) with 0.01 for –p-percentage. Data were visualized using ggplot (version 3.4.0) and ggprism (version 1.40.0) (Wickham 2009; Dawson 2021).

## Results

### *Nukadoko* formed a conservative microbiome

**Figure 2.**
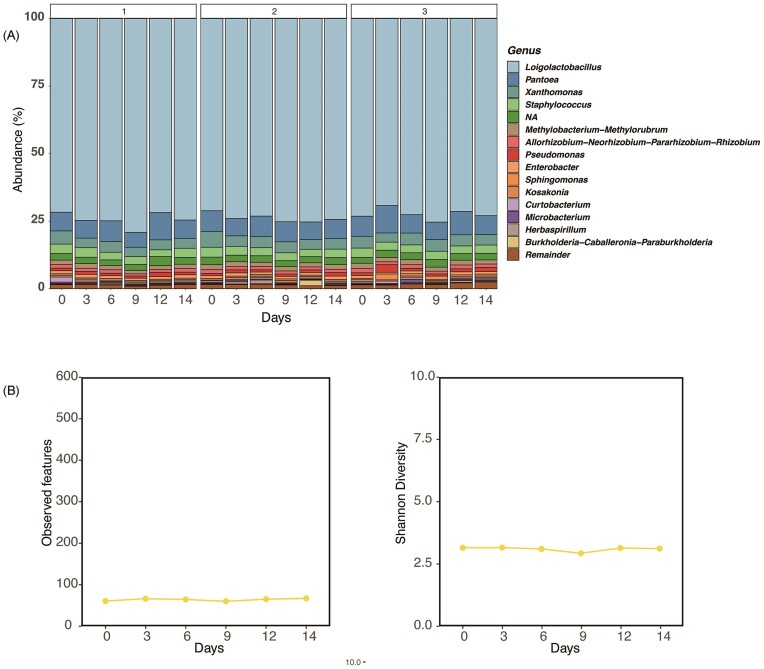
Changes in the microbial composition of the rice-bran beds. (A) Relative composition of the microbiome of rice-bran beds maintained by each participant over time. The top 15 genera are presented by their names, and the rest are grouped as the *remainder*. (B) Time-course changes in observed features and Shannon diversity index in microbial communities of rice-bran beds.

First, 16S rRNA amplicon sequencing was performed to investigate the extent to which the skin microbiome affected the rice bran beds. After removal of mitochondrial and chloroplast-derived reads, we obtained 18 114 reads at maximum, 13 053 reads at minimum, and 15 937 reads at the median for *Nukadoko* samples and 21 706, 41 949, and 32 996 reads for skin samples. Details of the reads generated from DADA2 are presented in Table S1. We did not observe substantial changes in the microbiome composition of *Nukadoko* over two weeks compared with that on day 1. Specifically, the *Loigolactibacillus* genus was predominantly abundant among all three participants and accounted for approximately 69%–79% of the relative abundance throughout the 14 days (Fig. 2A). *Pantoea* was the second most common genus, accounting for 5%–10% of the total. *Xanthomonas* and *Staphylococcus* were also identified on all the days. However, the trend of *Loigonolactobacillus* comprising much of the microbiome composition did not change. Shannon diversity index, as an alpha diversity indicator, and the observed features did not show any substantial variation. Shannon diversity index was approximately 6 consistently, and the observed features were approximately 50, showing daily and participant-specific variation, both slight (Fig. 2B).

### The skin microbiome varies from participant to participant

**Figure 3.**
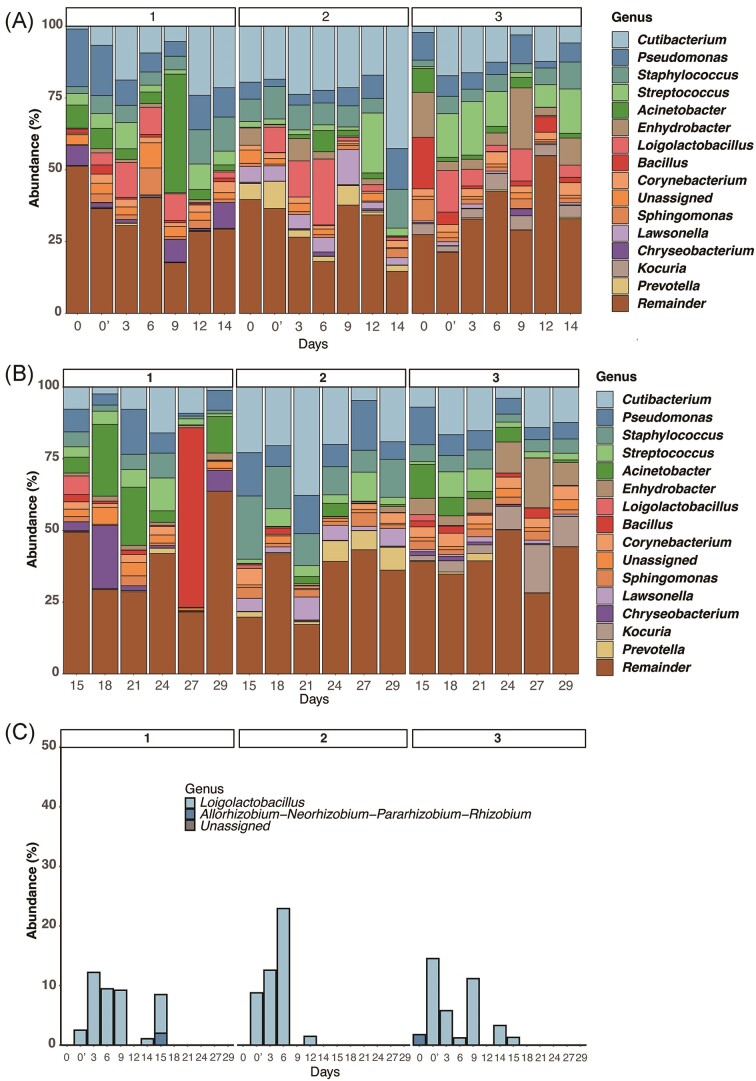
Changes in the microbial composition of the skin with the interaction of the rice-bran beds. (A) Relative composition of the skin microbiome of each participant over time from day 0 to day 14. The top 15 genera are presented by their names, and the rest are grouped as the *remainder*. (B) Relative composition of the skin microbiome without interaction with *Nukadoko* from each participant over time from day 15 to day 29. (C) The abundance of shared ASVs and their compositions. The X-axis shows days and the Y-axis indicates the proportion of shared ASVs in skin. 'Day 0' is before interaction, and 'Day 0’' is 6 h after interaction.

Participants stirred the bran and collected microbiomes from their palms using the swab method 6 h later by themselves. In contrast to the *Nukadoko* microbiome, the skin microbiome varied from participant to participant (Fig. 3A). Across the participants, *Cutibacterium, Pseudomonas, Staphylococcus*, and *Acinetobacter* were the most common genera. *Acinetobacter* was more abundant in Participant 1, while *Cutibacterium* was more consistently identified in Participant 2, and *Kocuria* was particularly identified in Participant 3 than in the other two participants. Participants spent two weeks maintaining *Nukadoko* with monitoring and were further observed for two weeks without contact with it (Fig. 3A, B).

We computed the shared ASVs to determine the extent to which microorganisms were transferred between *Nukadoko* and the participants’ palm skin. The abundances of the shared ASVs in skin samples are shown as a bar graph (Fig. 3C). Shared ASVs were not detected before stirring *Nukadoko* (Pre-interaction on Day 0: Day 0) except for subject 3 (1.77%). We confirmed that shared ASVs were positively detected on the skin throughout the first two weeks including post-interaction on Day 0 (Day 0’). The range of the shared ASVs was from 1.08% to 22.9%. The identified and shared ASVs were derived from *Loigolactibacillus, Allorhizobium-Neorhizobium-Pararhizobium-Rhizobium*, or *Unassigned* (Fig. 3C)*. Loigolactibacillus* which had become the dominant genus in Nukadoko, was detected as dominant in the shared ASVs of the skin microbiome of all participants. The shared ASVs were detected on subjects 1 and 3 on day 15. No further observations of the shared ASVs were made after day 15.

## Discussion

This study revealed that *Nukadoko*, at the late stage, formed an extremely conservative and stable microbial community. *Loigolactibacillus*, the dominant species of *Nukadoko*, was briefly transferred to the skin microbiome.

Out of the three stages of *Nukadoko*, the initial stage (before day 10), middle stage (day 10–30), and late stage (after day 30), the late stage was investigated in this study (Ono et al. 2014). Previous studies have investigated the stable expansion of the microbiome in rice bran beds by inoculating plain rice bran with a fermentation starter and maintaining the transition of the microbiome through the three stages (Sakamoto et al. 2011). However, most customers buy commercially available matured *Nukadoko* and ferment vegetables by soaking them. No research has yet been conducted on microbiome variation during the maintenance of this fermented food at the late stage. To the best of our knowledge, this is the first study to address this issue. The most important characteristic of *Nukadoko* is that it requires careful stirring with bare hands by caretakers. Because of this, *Nukadoko* is always at risk of the easy introduction of foreign and undesirable microbes. The skin microbiome can also harbor bacteria that cause food poisoning, such as *Staphylococcus* (Kadariya, Smith and Thapaliya 2014).

In this study, three different participants maintained *Nukadoko* at home, and the microbiome hardly fluctuated in any of the batches over two weeks. The genus *Loigolactibacillus* was the priority species for the *Nukadoko* investigated (Fig. 2A). Alternatively, *Nukadoko* and pickled vegetables with *Lactiplantibacillus plantarum* as the dominant species and extremely diverse microorganisms was previously reported (Ono et al. 2014; Sawada et al. 2021). One of the problems in this comparison is the reclassification of the *Lactobacillus* genus in 2020 (Zheng et al. 2020). A reanalysis of past studies is required to coordinate groups in *Nukadoko* based on their microbiome characteristics. Although absent in *Nukadoko* used in this study, *Halomonas* spp. found in the final product of pickled vegetables has been reported to contribute to the elevation of glutamate concentrations (Sawada et al. 2021). Microorganisms in *Nukadoko* may contribute to the formation of flavors, and the role of each microorganism should be thoroughly investigated in future studies. *Nukadoko* is a fermented food that is customizable and requires consideration of numerous parameters to identify its chemistry, including the ingredients to be utilized, the location of the fermentation, and the people who will produce it. Thus, developing a microbiome database of the fermented food can lead to safer and more flavorful fermentation.

Several studies have used shared ASVs, including bacterial ASV transmission analysis, to determine the extent to which microorganisms are shared between mothers and infants (Maqsood et al. 2019) and a survey on microorganisms in milk collected from several regions and seasons in China (Liang et al. 2022). In our study, *Nukadoko* was collected before stirring, and skin samples were collected 6 h after stirring. Therefore, *Nukadoko* and skin samples were used for pairwise shared ASVs analysis, allowing us to confirm the sharing rate on each day (Fig. 3C).

16S rRNA amplicon sequencing is becoming an increasingly useful and affordable technique for microbiome screening. However, it has become clear that the results vary depending on the DNA extraction method, type of universal primer utilized, and method of analysis (Keisam et al. 2016). Similarly, some studies have reported that sampling methods also affect the alpha diversity of skin microbiome (Bjerre et al. 2019). Therefore, to allow for variations owing to technical problems, the threshold for shared ASVs was set to 1% in this study. Shared ASVs are a valid calculation for identifying the microbial source of fermented foods but is limited by the shortcomings of 16S rRNA amplicon sequencing. To clarify the extent to which microorganisms have been transferred, it is necessary to detect cells at the single-cell level and comprehensively compare the results, using metagenomics. Another technical limitation of 16S rRNA amplicon sequencing is the inability to distinguish between live and dead bacterial cells. To assess the impact of bacteria more accurately, it is necessary to employ culturing or staining-based methods that provide higher resolution. Also, 16S rRNA amplicon sequencing only detects bacteria, whereas yeast has been reported in rice bran. Yeast plays an important role in the flavor of bran as it is responsible for ethanol fermentation. It is necessary to investigate the amount of yeast present in bran beds by ITS amplicon sequencing or metagenomic analysis.

Studies of skin microbiome transfer have been reported in the past that considered the results of microbiome transfer from different donors to participants over a 24-h timescale (Perin et al. 2019). This study suggests that the microbiome implanted in the donor is present for 24 h. Our data are consistent with this, as *Loigolactibacillus* was identified on day 15, even after the participant stopped touching the bran on day 14. The microorganisms may have different effects on the host in terms of the settlement, but even touching the bran bed may cause attachment for a short period.

We have previously investigated the emotional relationships between human maintaining *Nukadoko* and its microbiome using an interactive *Nukadoko* robot or *Nukabot* (Chen et al. 2021). In the context of human–computer interaction, we evaluated the process of participants gaining awareness of native microorganisms through vocal conversation. When *Nukadoko* was stirred daily at increasing rates, more conversation took place, and a higher sense of emotional care was generated among the participants. In this study, we revealed a bacterial transfer from *Nukadoko* to the human skin microbiome. However, bacterial communication can occur in bidirectional, from *Nukadoko* to human, and human to *Nukadoko*. Although there have been studies on the production of *Nukadoko*, none have addressed how much of the microbiome in *Nukadoko* comes or transfers from humans during its initial stages. Thus, much remains to be discovered about microbial-level interaction between humans and *Nukadoko* and human–food communication.

## Supplementary Material

fnad120_Supplemental_Files

## Data Availability

The datasets generated through 16S rRNA amplicon sequencing are available and deposited in the NCBI Sequence Read Archive (SRA) database under accession numbers DRR433234-DRR433293 and BioProject PRJDB14941.

## Code availability

q2-shared_asv is available through https://github.com/biota-inc/q2-shared_asv.
