# Supplementary figures and images for: Direct contact of fermented rice bran beds promotes food-to-hand transmission of lactic acid bacteria
Source: FEMS Microbiol Lett. 2023 Nov 13;370:fnad120. doi: 10.1093/femsle/fnad120 (PMC10697408; doi:10.1093/femsle/fnad120)

Supplementary Figure 1. Alpha rarefaction curve

**Supplementary Figure 1.**

**
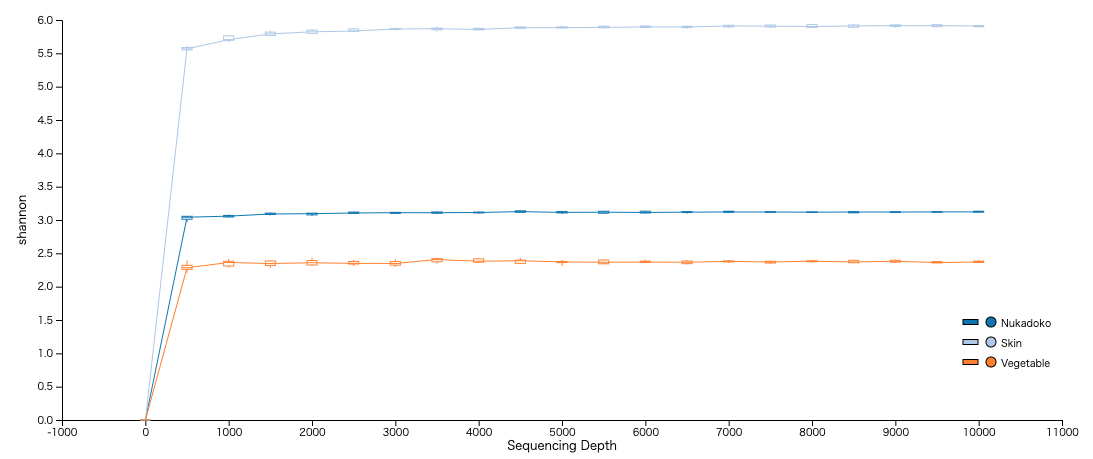
**

Supplement: fnad120_Supplemental_Files [file fnad120_supplemental_files.zip › Supplementary_figure.docx]
